# Giant, Bleeding, and Ulcerating Proliferating Trichilemmal Cyst, With Delayed Treatment Due to Coronavirus Outbreak: A Case Report and Review of the Literature

**DOI:** 10.3389/fsurg.2021.680160

**Published:** 2021-11-26

**Authors:** Cecilie Mullerup Kiel, Preben Homøe

**Affiliations:** Department of Otorhinolaryngology and Maxillofacial Surgery, Zealand University Hospital, Roskilde, Denmark

**Keywords:** trichilemmal cyst, proliferating trichilemmal cyst, pilar tumour, lipoma, case report, corona

## Abstract

We report a case of a large, ulcerating proliferating trichilemmal cyst in a 63-year-old woman, with clinical, radiological, macroscopic, and microscopic correlation. The outbreak of the Coronavirus pandemic delayed her treatment. We review the literature on proliferating trichilemmal cysts, which are relatively rare tumors, which generally are considered benign. However, we found a high rate of malign cases, which stresses the importance of rapid surgical excision and histological diagnosis. Even though our proband had delayed treatment, the tumor did not transform to a malignant form.

## Introduction

Proliferating trichilemmal cysts (PTCs) are relatively rare tumors that may appear all over the body but most frequently on the scalp in middle-aged women (1). PTCs occur in a benign and malignant form, but the differentiation between malignant PTC and benign PTC has been debated, implying that all PPT should be treated with the expectation that it could transform into a malignant tumor (2). There are no absolute clinical criteria that can differentiate between benign and malign PTC, why surgery is necessary to give a correct histopathological diagnose. The malignant form may metastasize (3).

We here report the case of a 63-year-old woman with a large PTC, where surgical treatment was delayed due to the outbreak of the Coronavirus pandemic and a review of the relevant literature on PTCs.

## Case Description

**Figure 1:**
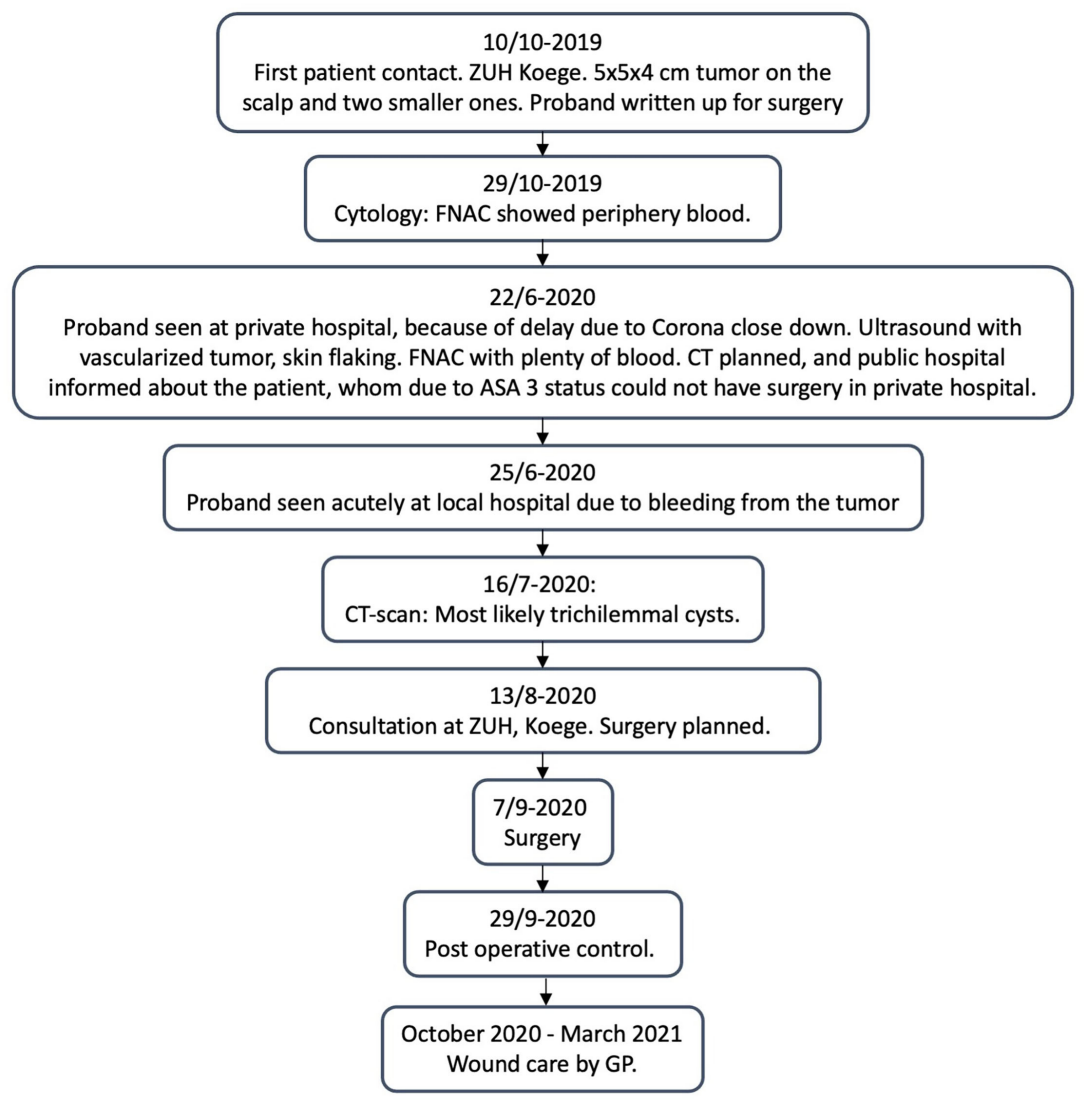
Timeline.

**Figure 2:**
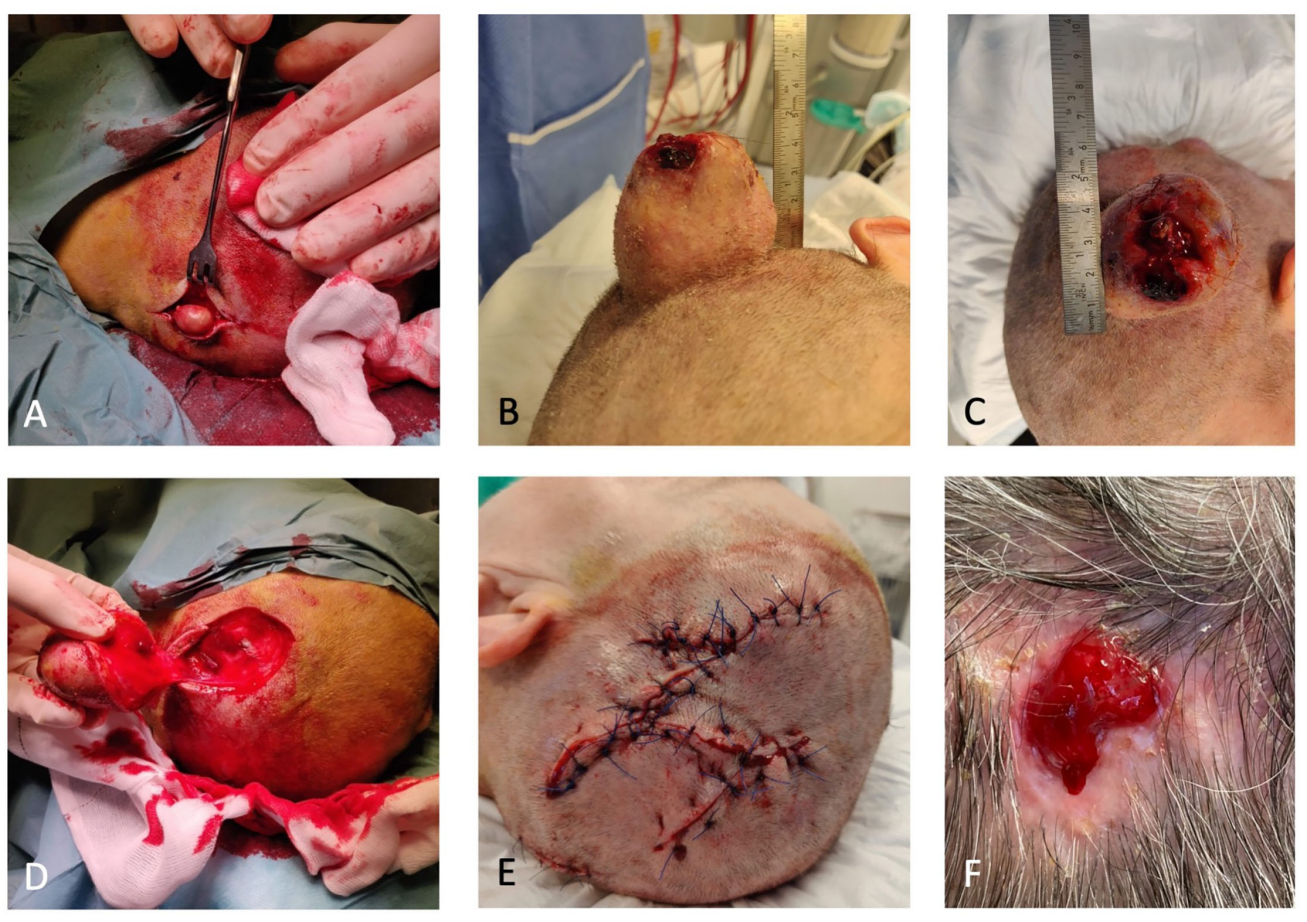
**(A)** One of the two smaller trichilemmal cysts (TCs). **(B)** Proliferating trichilemmal cyst (PTC) peroperative. **(C)** PTC with an ulcerating center. **(D)** Defect after removal of PTC. **(E)** Closure with local fasciomusculocutaneous flaps. **(F)** Small wound 6 months after primary closure.

The proband is a 63-year-old woman, with ASA 3 status, who rarely left her home due to sequelae from previous apoplexy. She had some minor tumors on the scalp for many years, for which she had not seen a doctor because they did not bother her. When one of them grew within a few months, she went to her General Practitioner (GP) for treatment. Her GP referred her, to the Department of Otorhinolaryngology and Maxillofacial Surgery at Zealand University Hospital (ZUH), Koege, in October 2019, for a lipoma on the scalp measuring approximately 5 cm in diameter (for timeline see Figure 1). She had no B-symptoms. There was no familiar history of tumors, besides her dad, who had a lipoma on the scalp removed. At first consultation in October 2019, a sizeable painless tumor of 4 × 5 × 5 cm and two smaller tumors were found on the scalp (Figure 2B). The tumors were movable from the underlying structures and resembled lipomas. Surgery was recommended.

## Diagnostic Assessment

**Figure 3:**
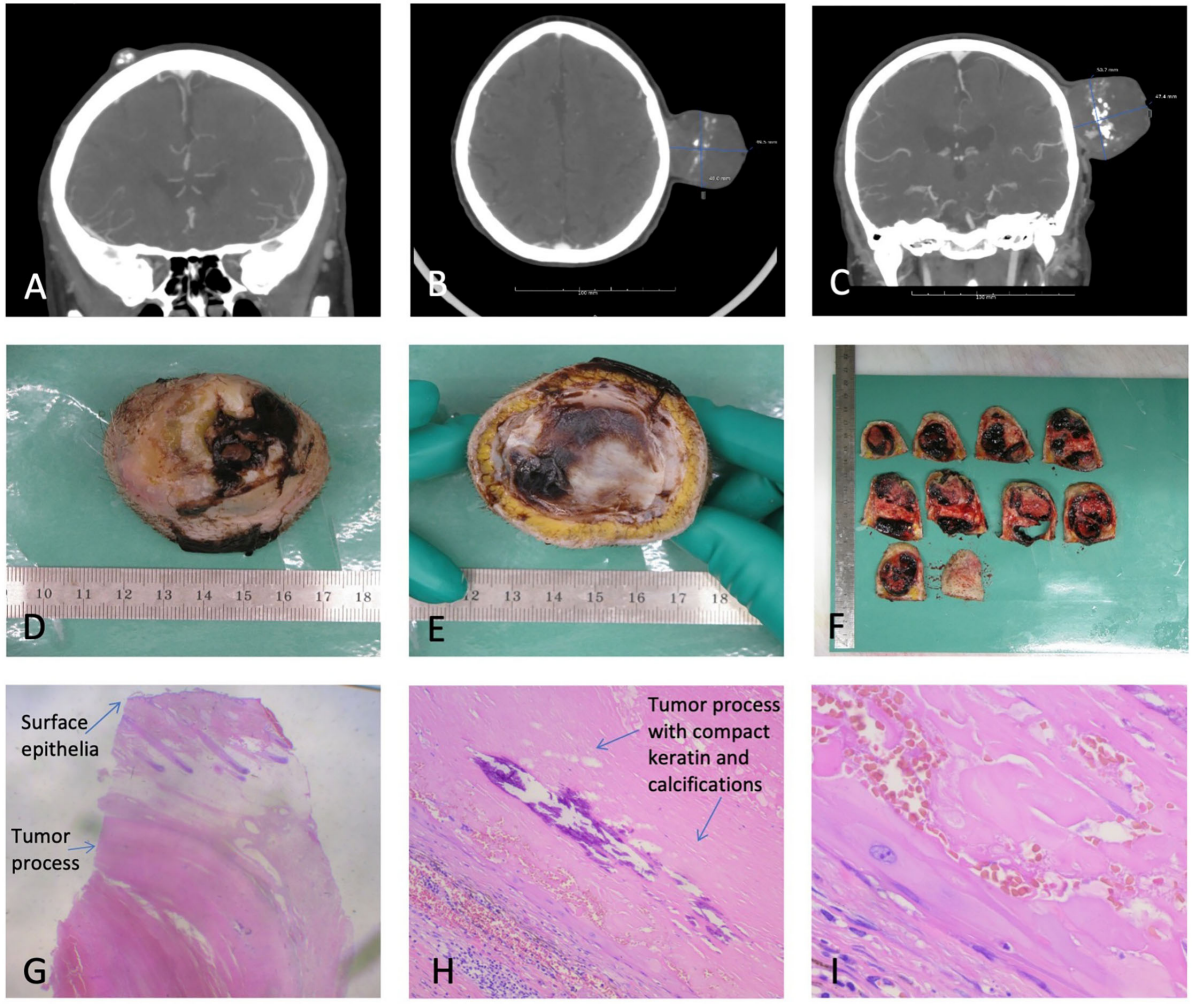
**(A)** One of the two smaller TCs. **(B)** PTC axial view. **(C)** PTC coronal view. **(D,E)** Macroscopy of the PTC. **(F)** Macroscopic view of sliced PTC. **(G)** Microscopy of PTC, depicting surface epithelia and a tumor process. **(H)** Microscopy depicting the compact keratin and calcifications. **(I)** Microscopy with squamous epithelial cells without a granular cell layer.

Ultrasound in October 2019 showed a slightly inhomogeneous subcutaneous process and fine needle aspiration cytology (FNAC) with blood cells. In June 2020, the ultrasound at a private hospital found a vascularized tumor, and a needle puncture gave plenty of blood. A CT scan from July 2020 showed a marked progression of an extracranial tumor compared to a CT cerebrum, which was performed because the proband had cerebral apoplexy in 2012 (Figure 3). The process was found inhomogeneous and contained popcorn-shaped calcifications and multiple small vessels after intravenous contrast. The process was close to the temporalis muscle and the subcutaneous connective tissue but without skull ingrowth. Two smaller similar processes were present: one occipital measuring 15 mm and one frontal measuring 12 mm.

Because of the probands ASA 3 status, she was referred back to the ZUH, Koege, for surgery. She had trouble sleeping due to the anatomy of the tumor and fear of bleeding from the tumor. The proband underwent complete surgical resection of all three lesions. We reconstructed the extensive scalp defect caused by the tumor resection with local fasciomusculocutaneous flaps (Figure 2).

Macroscopic examination (Figure 3) showed a skin-covered tumor with craterlike ulceration, measuring 47 mm in height and 50 × 50 mm at the base with the fascia intact. The tumor contained cystic and solid areas, with bleeding, coagulation, and calcifications. Microscopy revealed a well-defined epidermis with central ulceration. The dermis was filled by a well-defined tumor consisting of multiple, size-varying, and confluent lobuli with fibrosis in between. The lobuli were clad with the squamous epithelium of trichilemmal type, without the stratum ganulare, and filled out by compact keratin with a varying degree of calcification and bleeding. Focal necrosis was present. The tumor mainly consisted of cyst content. Atypia was not present. The histopathological diagnosis was skin with PTC, without any malignant signs. The two smaller tumors were TCs.

At postoperative control, small central necrosis was present. Due to the long distance to the hospital, the proband wished further wound care to be at her own GP. The wound became infected with Staphylococcus Aureus and was treated with antibiotics. The wound was almost healed by March 2021, where only a minor defect was present as seen in Figure 2F.

## Discussion

The proliferating trichilemmal cyst was first described as a proliferating epidermoid cyst by Wilson Jones in 1966 (4). Just months after, McGraven and Binnington published their study on electron microscopy of sebaceous cysts, which established the keratinizing nature of cellular maturation derived from the piliary apparatus, distinguishing it from epidermoid cysts, and proposed the name pilar cyst. The name “trichilemmal cyst” was finally suggested by Pinkus in 1969 (5), as he “put the myth of the sebaceous cyst to rest.”

### Epidemiology and Clinical Presentation

The common pilar cyst (or TC) occurs in 5–10% of the population, of which 2% become PTCs (6). PTC is often found on the scalp (1, 7, 8). Our literature search confirmed this, but we also found a case on the elbow, three cases on the finger, three on the mammae, one on the eyelid, and other locations as well (9–17). Because of this, a location outside the scalp should not rule out PTC when no pathology is present.

The proliferating trichilemmal cyst appears clinically as a subcutaneous nodule. It can resemble a cyst or lipoma, and the skin can be atrophic or, as in our case, flaking and ulcerated. PTC is often confused with squamous cell carcinoma (SCC). Other differential diagnoses are epidermoid cyst, keratoacanthoma, sweat gland tumor, basal cell carcinoma, angiosarcoma, pilomatrixoma, dermatofibrosarcoma protuberans, and cylindroma (18). TCs often occur as sporadic lesions, but they are also found in hereditary settings with the autosomal dominant transmission. In our case, the parents of the proband did not have confirmed TCs, but the father of the proband had had a forehead tumor removed. Seidenari et al. studied 149 cases of TC and found 16.1% to arise in probands with at least one first-degree relative with a diagnosis of TCs (19), and found 49% of probands under the age of 45 reported a positive family history. Therefore, when a PTC is present in younger persons, one should look at a possible geneticdisposition.

### Histopathology

Fine needle aspiration cytology helps the surgeon differentiate between malignant and benign lesions. Definitive diagnosis can be challenging on FNAC from a cystic lesion, as enough cells are required, and FNAC from PTC is, therefore, often misdiagnosed. This is supported by the cytological study by Shet et al., who found TCs to yield more keratinous debris and sparse to no epithelia on FNAC (20). TCs should be suspected when small basaloid or squamoid cells in paucicellular aspirate are seen, especially if the calcification is present. However, Shet et al. also acknowledged the risk of missing focal malignant change, which is more common in FNAC compared to histology.

The proliferating trichilemmal cyst is composed of variable-sized lobules, which macroscopically give the PTC a honeycomb appearance, with small cysts filled with keratin material (21). Microscopically, the PTC is composed of proliferating lobules of squamous epithelium, with multiple central areas of trichilemmal keratinization and the formation of homogenous keratin cysts (6). The trichilemmal keratin arises in the stratified epithelium of the isthmus of the outer root sheath of the hair (5); squamous epithelium undergoes rapid keratinization without the formation of a granular cell layer. This produces a cyst wall with a direct transition from the spinal layer to the stratum corneum without the normal granular layer in between. This abrupt keratinization helps differentiate the TC from an epidermoid cyst, which still retains the granular layer (22). However, differentiation of SCC and PTC can be more challenging, as tumor cells in the PTC might show nuclear atypia, mimicking SCC (13). The presence of trichilemmal keratinization and lack of a granular layer is generally accepted as histologic hallmarks of TCs (18). Foci of calcification, necrosis, and hyalinization may be present (23). Variable inflammatory reactions, including foreign body giant cells, are seen in the stroma, which is usually fibrous.

Immunohistochemistry has been examined widely in different cystic tumors, and PTCs have been found positive for keratin markers K10 and K17 (24). Immunohistochemistry can potentially assist in subtyping PTC, for example, by using the proliferation markers Ki-67 and p53 (25). CD34 expression supports the outer root sheath origin of the tumor, and its presence might help differentiate between malignant PTC and SCC, as malignant tumors show little to no immunoreactivity to CD34 (18, 26).

### Radiology

Proliferating trichilemmal cysts have been studied with MRI, ultrasound, and CT (13, 23, 25, 27, 28). A CT scan of a 54-year-old woman with a PTC showed a well-enhancing wall of variable thickness with multiple speckled calcifications (23), with no evidence of extracapsular spread, much equal to the CT findings in our case. A study of 54 TCs (of which four were PTCs) found no doppler sonography (28), contrary to the findings of our case, where an ultrasound showed a vascularized tumor. This discrepancy could be because our case was a PTC, and the study by He et al. only examined four PTCs; however, Miyachi neither found increased blood flow on ultrasound in a PTC (27)—further studies on the validity and characteristics of ultrasound are missing. He et al. also found that 72% were hyperechoic masses, 89% were heterogeneous, helping differentiate the PTC from lipomas. About 65% of the tumors had internal calcification. Posterior enhancement was also common, as seen in 84% of the cases (28). Examined by MRI, the PTC shows heterogeneous signals on T2-weighted images (18). Lymphadenopathy may be detected clinically or by CT, MRI, or ultrasound, suggesting potential metastases.

### Treatment/Management

Adequate treatment of proliferating trichilemmal cysts includes surgical excision and skilled histopathological examination of the excised tumor for proper diagnosis. When malignancy is present, a 1-cm margin is recommended (29). Mohs surgery has also been used in recent years (30). Since clinical behavior with rapid growth might not correlate with disease progression, a histopathological diagnosis is essential to secure correct treatment and prevent a recurrence. If an extensive inoperable disease is present, radiotherapy is possible. Two cases exist in the literature: one 93-year-old man with good oncological and cosmetic results (31), and one younger woman with disfiguring PTC of the scalp, with good cosmesis and no signs of regrowth (32).

### Prognosis

**Figure 4:**
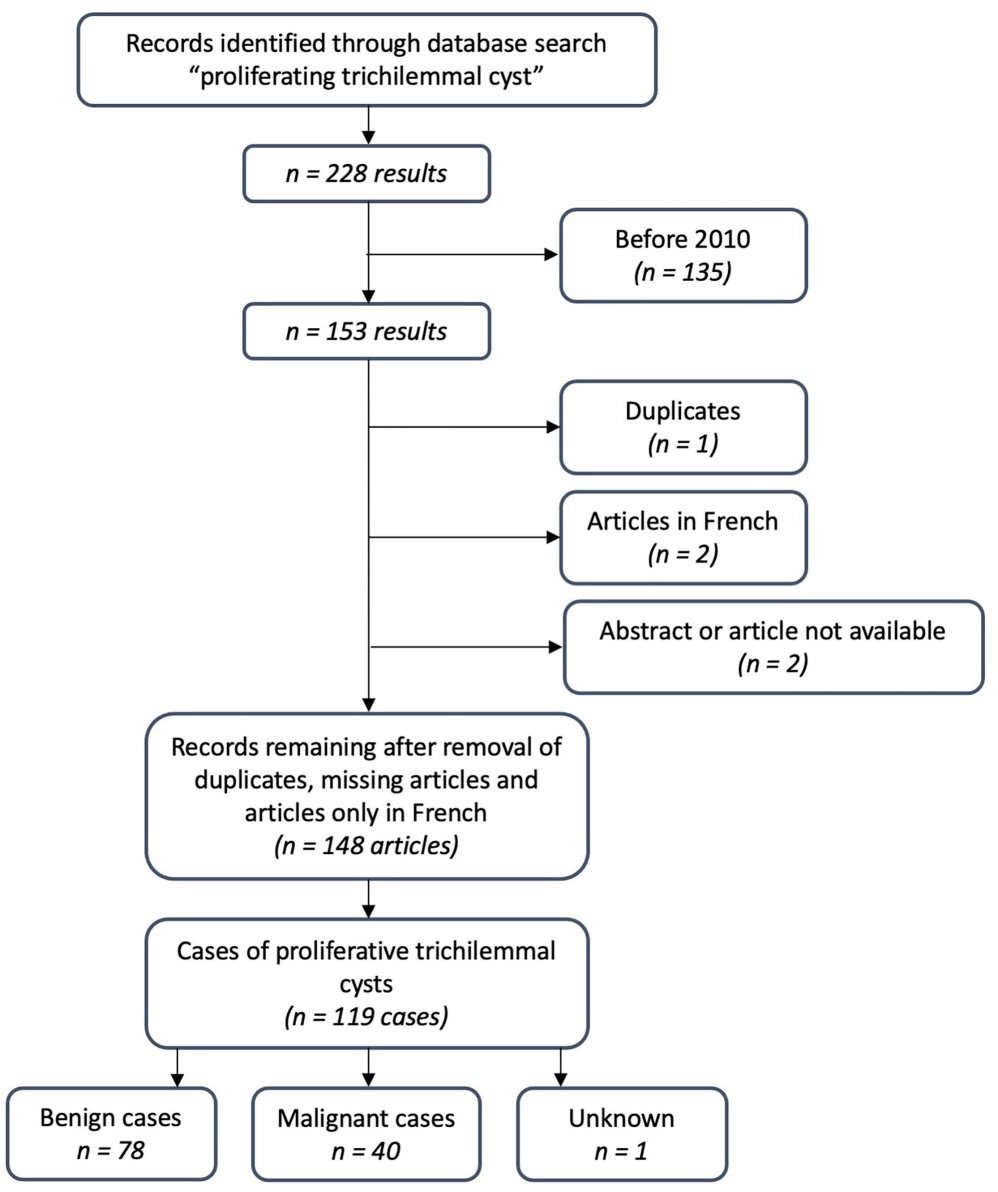
A flowchart of literature search.

Typically, TCs are present for years as “simple cysts” before they enlarge over months to years and yield disfiguring tumors that might ulcerate. According to Brownstein (33), trauma and inflammation may cause a TC to develop into a PTC. Brownstein compared 50 PTCs with “ordinary” TCs and found that, occasionally, probands had ordinary TCs and PTCs on their scalp (33), which was also the case for our proband. Generally, PTCs are recognized as benign but can be both locally aggressive and malign. The case of a 29-year-old woman with an intracranial component and lung metastasis (3) exemplifies the potential for a deathly path of PTC. Ye et al. proposed to classify PTC into benign, locally aggressive, and malignant, based on the correlation between histologic features with tumor behavior (7). A metanalysis found local recurrence to be 3.7% after wide local excision (18). We did a PubMed search on “proliferating trichilemmal cyst,” including articles from 2010 to 2021, revealing 119 cases, of which 40 were malignant (Figure 4). This finding suggests that the risk of transformation to a malignant tumor is higher than previously stated.

## Conclusion

A proliferating trichilemmal cyst is a cyst derived from the more common TC and should be considered when rapid growth occurs. Histological diagnosis might not correlate with the clinical course, and surgical excision should not be delayed because of the risk of malignant transformation. Even when benign, a larger tumor will only complicate surgical reconstruction of the area. The true incidence of malignant transformation of PTC is currently unknown but thought to be very rare (34). Based on our literature search and a review by Ye et al., malignancy may occur in more than 30% of PTCs (7). There is, however, a risk of publication bias. This relatively high incidence of malignancy reported further stresses the importance of surgical excision and histological diagnosis. In times with the Corona pandemic, where theaters have been closed down in many countries for non-acute or non-cancer surgeries, we propose that surgeons prioritize probands with PTC due to the risk of malignant transformation.

## Data Availability Statement

The original contributions presented in the study are included in the article/supplementary material, further inquiries can be directed to the corresponding author/s.

## Ethics Statement

Written informed consent was obtained from the individual(s) for the publication of any potentially identifiable images or data included in this article.

## Author Contributions

CK: general script, literature search, gathering information, and production of figures. PH: specialist consulting, rewriting and revisions, consent from the patient, and help with submission. All authors contributed to the article and approved the submitted version.

## Conflict of Interest

The authors declare that the research was conducted in the absence of any commercial or financial relationships that could be construed as a potential conflict of interest.

## Publisher's Note

All claims expressed in this article are solely those of the authors and do not necessarily represent those of their affiliated organizations, or those of the publisher, the editors and the reviewers. Any product that may be evaluated in this article, or claim that may be made by its manufacturer, is not guaranteed or endorsed by the publisher.
