# Corrigendum: Giant, Bleeding, and Ulcerating Proliferating Trichilemmal Cyst, With Delayed Treatment Due to Coronavirus Outbreak: A Case Report and Review of the Literature

**DOI:** 10.3389/fsurg.2022.865956

**Published:** 2022-05-11

**Authors:** Cecilie Mullerup Kiel, Preben Homøe

**Affiliations:** Department of Otorhinolaryngology and Maxillofacial Surgery, Zealand University Hospital, Roskilde, Denmark

**Keywords:** trichilemmal cyst, proliferating trichilemmal cyst, pilar tumour, lipoma, case report, corona

In the original article, the age of the woman in the case study was wrongly written as 76. Instead, the correct age should be 63.

Sections which have been changed:

We report a case of a large, ulcerating proliferating trichilemmal cyst in a **63**-year-old woman, with clinical, radiological, macroscopic, and microscopic correlation. The outbreak of the Coronavirus pandemic delayed her treatment. We review the literature on proliferating trichilemmal cysts, which are relatively rare tumors, which generally are considered benign. However, we found a high rate of malign cases, which stresses the importance of rapid surgical excision and histological diagnosis. Even though our proband had delayed treatment, the tumor did not transform to a malignant form.

**Introduction**

Proliferating trichilemmal cysts (PTCs) are relatively rare tumors that may appear all over the body but most frequently on the scalp in middle-aged women (1). PTCs occur in a benign and malignant form, but the differentiation between malignant PTC and benign PTC has been debated, implying that all PPT should be treated with the expectation that it could transform into a malignant tumor (2). There are no absolute clinical criteria that can differentiate between benign and malign PTC, why surgery is necessary to give a correct histopathological diagnose. The malignant form may metastasize (3).

We here report the case of a **63**-year-old woman with a large PTC, where surgical treatment was delayed due to the outbreak of the Coronavirus pandemic and a review of the relevant literature on PTCs.

**Case description**

The proband is a **63**-year-old woman, with ASA 3 status, who rarely left her home due to sequelae from previous apoplexy. She had some minor tumors on the scalp for many years, for which she had not seen a doctor because they did not bother her. When one of them grew within a few months, she went to her General Practitioner (GP) for treatment. Her GP referred her, to the Department of Otorhinolaryngology and Maxillofacial Surgery at Zealand University Hospital (ZUH), Koege, in October 2019, for a lipoma on the scalp measuring approximately 5 cm in diameter (for timeline see Figure 1). She had no B-symptoms. There was no familiar history of tumors, besides her dad, who had a lipoma on the scalp removed. At first consultation in October 2019, a sizeable painless tumor of 4x5x5 cm and two smaller tumors were found on the scalp (Figure 2B). The tumors were movable from the underlying structures and resembled lipomas. Surgery was recommended.

The authors apologize for this error and state that this does not change the scientific conclusions of the article in any way. The original article has been updated.

## Publisher's Note

All claims expressed in this article are solely those of the authors and do not necessarily represent those of their affiliated organizations, or those of the publisher, the editors and the reviewers. Any product that may be evaluated in this article, or claim that may be made by its manufacturer, is not guaranteed or endorsed by the publisher.

